# Decellularized Annulus Fibrosus Matrix/Chitosan Hybrid Hydrogels with Basic Fibroblast Growth Factor for Annulus Fibrosus Tissue Engineering

**DOI:** 10.1089/ten.tea.2018.0297

**Published:** 2019-12-12

**Authors:** Chen Liu, Zhongxing Jin, Xin Ge, Yu Zhang, Hongguang Xu

**Affiliations:** Department of Orthopaedics, Yijishan Hospital of Wannan Medical College, Wuhu, China.

**Keywords:** decellularized annulus fibrosus matrix, chitosan, annulus fibrosus-derived stem cells, hydrogels

## Abstract

Low back pain caused by degenerative disc disease affects many people worldwide and brings huge economical burden. Thus, attentions have focused on annulus fibrosus (AF) tissue engineering for treatment of intervertebral disc degeneration. To engineer a functional replacement for the AF, it is important to fabricate scaffolds that mimic the structural and mechanical properties of native tissue. AF-derived stem cells are promising seed cells for AF tissue engineering due to their tissue specificity. In the present study, decellularized AF matrix (DAFM)/chitosan hybrid hydrogels were fabricated using genipin as a crosslinker. AF stem cells were cultured on hydrogel scaffolds with or without basic fibroblast growth factor (bFGF), and cell proliferation, morphology, gene expression, and AF tissue synthesis were examined. Overall, more *collagen-I*, *collagen-II*, and *aggrecan* were secreted by AF stem cells grown on hydrogels with bFGF compared to those without. These results support the application of DAFM/chitosan hybrid hydrogels as an appropriate candidate for AF tissue engineering. Furthermore, incorporation of bFGF into hydrogels promoted AF-related tissue synthesis.

**Impact Statement:**

The investigation of annulus fibrosus (AF)-related tissue secretion and gene expression in extracellular matrix (ECM) of AF-derived stem cells (AFSCs) provided theoretical and practical basis for the choice of scaffold materials and growth factors for AF tissue engineering. The innovations of the present work are obvious. First, AFSCs were used because they are more easily differentiated into AF cells, thereby producing more AF-related ECM. Second, the decellularized AF matrix (DAFM) was derived from native AF tissue, but had reduced immunogenicity after decellularization. Furthermore, the DAFM structure mimicked the fibrous network of actual AF tissue, which was advantageous to AFSC adhesion and growth. Third, basic fibroblast growth factor was successfully incorporated into the DAFM, showed gradual sustained release, and effectively promoted production of AF tissue ECM factors collagen-I, collagen-II, aggrecan, and glycosaminoglycan.

## Introduction

Degenerative disc disease is a main cause of low back pain in middle-aged and elderly people, which seriously affects quality of life.^1^ Medical costs and annual economic losses associated with this disease are reported to be as high as $16 billion in the United States alone.^2^ At present, treatment of degenerative disc disease includes both conservative and surgical approaches. While surgical treatment, such as vertebral fusion, can significantly improve symptoms of low back pain in patients,^3^ postoperative biomechanical changes of the spine typically occur that can accelerate degeneration of adjacent segments, leading to more serious spinal disorders.^4^ Recently, cell-based tissue engineering for the intervertebral disc has been shown to be a promising option for treatment of this disease.^5^

Anatomically, the intervertebral disc is composed of a central nucleus pulposus circumferentially surrounded by the annulus fibrosus (AF). The nucleus pulposus resists compressive load due to its high hydration and large amounts of hydrated proteoglycan, whereas the AF is a complex fibrocartilage tissue with 15 to 25 concentric lamellae, which are held together by collagen fibers.^6,7^ The integrity of the AF structure and function is essential to maintaining the proper morphological position of the nucleus pulposus as well as physiological pressure within the disc.^8^

A number of attempts have been made to engineer the AF using synthetic or natural polymers as scaffold materials. However, the collagen and glycosaminoglycan (GAGs) content within the matrices of these studies were much lower than that in the actual tissue, causing the eventual failure of these attempts.^9–11^ Therefore, new types of biomaterials that enable unhindered cellular proliferation and matrix production need to be developed. Indeed, an ideal scaffold should have good biocompatibility, a proper *in vivo* degradation rate, as well as a similar matrix composition, structure, and mechanical properties as natural tissue.^12^ With the development of decellularization technology, native tissue scaffolds have received more and more attention in recent studies by providing the same or similar microenvironment for seeded cells as existing extracellular matrix (ECM) *in vivo.*^13^

Decellularized matrices are produced by removing cellular components of the organization using chemical, enzymatic, or mechanical methods while retaining other components of the ECM. Decellularization not only maintains the original organization and biological function of the matrix that is necessary for cell adhesion, expansion, and differentiation but also significantly reduces autoimmunity.^14,15^ Although decellularization has been applied to human tissues for tendon reconstruction, skin grafting, and vasculature engineering, there have been few studies related to use of a decellularized AF matrix (DAFM) to engineer AF tissue.^16^

Despite the advantages of using DAFM, it has poor mechanical properties that may be a limiting factor when engineering AF tissue. Thus, investigation into other materials that could be incorporated to help circumvent this limitation and further benefit AF tissue growth is important. In particular, chitosan has been shown to have good mechanical properties, such as stiffness,^17^ and can be crosslinked by a variety of agents, including genipin, a natural crosslinker with low toxicity, and anti-inflammatory effects.^18–20^

Moreover, addition of growth factors which stimulate and support tissue growth by regulating the proliferation, differentiation, and ECM metabolism of seed cells has been shown to be of great benefit. Growth factors used in AF tissue engineering have included basic fibroblast growth factor (bFGF), transforming growth factor (TGF)-β1, TGF-β3, and insulin-like growth factor (IGF)-1.^21–24^ Specifically, bFGF has not only been shown to stimulate fibroblastic differentiation of mesenchymal stem cells (MSCs) but also MSC proliferation and self-renewal.^25^ However, like most growth factors, bFGF has a very short plasma half-life (usually 90 s),^26^ thereby requiring repeated local administration for optimal effect. Hence, incorporating growth factors into hydrogels is an attractive way of ensuring their continued local release and effect on seeded cells.^27^

In the present study, DAFM/chitosan hybrid hydrogels with bFGF incorporated into the matrix were fabricated with genipin crosslinking. Using AF-derived stem cells (AFSCs) to seed hydrogels, cellular proliferation, morphology, gene expression, and AF-related tissue synthesis were evaluated. It is hypothesized that more AF-like ECM will be produced by continuous release of bFGF, making our DAFM/chitosan hybrid hydrogels even more suitable for engineering AF tissue.

## Materials and Methods

### Fabrication of DAFM/chitosan hybrid hydrogels

The Institutional Review Board of Wannan Medical College (Wuhu, China) approved all animal procedures. The nucleus pulposus and lateral fascia adipose tissue around the AF of five New Zealand white rabbits (6–8 weeks-old) were removed and washed three times with phosphate-buffered saline (PBS). The AF tissue was cut into 1 × 1 × 1 mm pieces, placed in a mortar, stiffened by adding liquid nitrogen, and ground to a course powder with a pestle. Decellularization began by incubating the ground tissue with 0.25% trypsin solution in a constant temperature shaker at 37°C for 24 h. The trypsin solution was changed every 4 h, and the mixture was washed three times with PBS. Then, ribozyme solution (10 mmol/L Tris-HCl containing 50 U/mL DNase and 1 U/mL RNase, pH = 7.5) was added, and tissue was further digested for 12 h at 37°C in a constant temperature shaker. Finally, 1% Triton X-100 was added for 24 h followed by washing with PBS six times for 8 h each. The precipitate was dissolved in 3% acetic acid, and the DAFM solution was stored in a refrigerator at 4°C.

Hybridization began by dissolving chitosan powder (1.5 g) in 3% acetic acid (100 mL) and genipin powder (0.1 g) in 75% ethanol (10 mL). Then, 3 mL of the chitosan solution was mixed with 3 mL of the DAFM solution and 1 mL of 1% genipin solution by stirring. Hybrid hydrogels were formed by adding 200 μL of the DAFM/chitosan/genipin solution to 24-well plates and then freeze-drying for 24 h. Dried hydrogel samples were sputter-coated with gold and visualized under the scanning electron microscope (SEM, S-4800; Hitachi Co. Ltd., Japan) at 3 kV accelerating voltage. The contact angle of the scaffold was measured by the deionized water bubble method using a KRÜSS DSA25 contact angle tester (KRÜSS Co., Germany). The average was taken from five samples.

### bFGF incorporation into hydrogels and release kinetics

bFGF (2 μg; ABclonal Biotechnology Co., Ltd.) was dissolved in 333 μL of 5 mM Tris (pH 7.6) containing 0.1% bovine serum albumin (Sigma-Aldrich, St. Louis, MO), mixed with 7 mL of DAFM/chitosan solution and then formed into hydrogels as described above. To study bFGF release kinetics, DAFM/chitosan hybrid hydrogels containing bFGF were placed in 30 mL of PBS and incubated at 37°C, and samples of the medium were collected at various time points. The concentration of free bFGF in the medium was quantified by enzyme-linked immunosorbent assay (ELISA) kit (ABclonal Biotechnology Co., Ltd.) according to the manufacturer's instructions. Absorbance was read on a microplate reader (BioTek Instruments) at 450 nm.

### AFSC preparation and hydrogel seeding

AFSCs from a sixth New Zealand rabbit (6–8 weeks-old) were isolated as previously described.^28^ In brief, AF samples were isolated from intervertebral disc as described above, minced, and digested in low-glucose Dulbecco's modified Eagle's medium (DMEM) for 6 h with 150 U/mL collagenase I (Catalog no. C0130; Sigma-Aldrich). Samples were centrifuged at 1000 rpm for 10 min and cell pellets resuspended in low-glucose DMEM supplemented with 20% fetal bovine serum, 100 U/mL penicillin, and 100 mg/mL streptomycin and plated in 100-mm tissue culture dishes. Cells were maintained in a humidified incubator at 37°C with 5% CO_2_. The medium was changed every other day until subconfluence, and then cells were harvested using 0.25% trypsin-EDTA. Ninety-six-well plates containing DAFM/chitosan hybrid hydrogels with and without bFGF were seeded with second passage AFSCs (2 × 10^3^ cells/well) and cultured as before. AFSCs seeded on the culture plate plastic without hydrogels (2 × 10^3^ cells/well) were used as a control.

### AFSC proliferation and morphology

CCK-8 assay was used to determine AFSC viability on hydrogels. On days 1, 3, 5, and 7 of hydrogel culture, wells were washed twice with PBS before adding 100 μL of PBS and 10 μL of CCK-8 assay reagent (Dojindo Co., Japan) to each and incubating for 2 h. The absorbance at 450 nm was measured using a microplate reader to determine cell viability.

AFSC morphology was detected by cytoskeleton staining and SEM analysis after culturing for 3 days on scaffolds. For cytoskeleton staining, cells on scaffolds were rinsed with PBS twice, fixed in 4% paraformaldehyde for 15 min, permeabilized with 0.1% Triton X-100 for 5 min, and then stained with fluorescein isothiocyanate-phalloidin and 4′,6-diamidino-2-phenylindole for F-actin and nuclei detection, respectively. After washing with PBS, the morphology of cells from different scaffolds was visualized by fluorescence microscope (Zeiss Axiovert 200; Carl Zeiss, Inc., Thornwood, NY). For SEM analysis, cells on scaffolds were rinsed with PBS, fixed with 2.5% glutaraldehyde for 2 h, and rinsed with deionized water three times. Samples were then dehydrated using gradient concentrations of ethanol from 50% to 100% for 10 min each. Dried samples were sputter-coated with gold and visualized by SEM at an accelerating voltage of 3 kV.

### Gene expression

AFSCs gene expression was analyzed using real-time quantitative polymerase chain reaction (RT-qPCR) after 2 weeks of hydrogel culture. Total RNA was extracted using the TRIzol isolation system (Invitrogen) following the manufacturer's protocol. cDNA was synthesized using the Revert-Aid™ First-Strand cDNA Synthesis Kit (K1622; Fermentas) and oligo(dT) primers for 60 min at 42°C on a reverse transcription PCR system (Eastwin Life Science, Beijing, China). RT-qPCR was performed with a Bio-Rad CFX96™ Real-Time System using the SsoFast™ EvaGreen Supermix Kit (Bio-Rad). Primer sequences for *collagen-I*, *collagen-II*, *aggrecan*, and *GAPDH* (internal control) are listed in Table 1. The relative expression levels were calculated using the 2^−ΔΔCt^ method and normalized to the internal control.

**Table 1.** The Sequence of the Genes

Gene	Sequence	Accession number
Collagen-I	Forward: 5′-CTGACTGGAAGAGCGGAGAGTAC-3′	AY633663
	Reverse: 5′-CCATGTCGCAGAAGACCTTGA-3′	
Collagen-II	Forward: 5′-AGCCACCCTCGGACTCT-3′	NM_001195671
	Reverse: 5′-TTTCCTGCCTCTGCCTG-3′	
Aggrecan	Forward: 5′-ATGGCTTCCACCAGTGCG-3′	XM_002723376
	Reverse: 5′-CGGATGCCGTAGGTTCTCA-3′	
GAPDH	Forward: 5′-ACTTTGTGAAGCTCATTTCCTGGTA-3′	NM_001082253
	Reverse: 5′-GTGGTTTGAGGGCTCTTACTCCTT-3′	

### ECM content

After culturing AFSCs on hydrogels for 2 weeks, samples of the medium were collected to measure the content of collagen-I, collagen-II, and aggrecan using ELISA kits (Jianglai Bio, China) following the manufacturer's protocol. Total GAG content was quantified by 1,9-dimethylmethylene blue staining using a commercially available kit (GMS 19239.2; Genmed Scientifics, Inc.).^29^ DNA content was determined using DNA-binding fluorochrome Hoechst 33258 dye. The collagen-I, collagen-II, aggrecan, and GAGs content were normalized to DNA content.

## Statistical analysis

The data are expressed as means ± SD. The statistical analyses were performed using SPSS 19.0 software. Kruskal–Wallis nonparametric analysis of variance was used to analyze the gene expression levels and collagen-I, collagen-II, and aggrecan content. *p* < 0.05 was regarded as significant.

## Results

### Scaffold characterization

**FIG. 1.**
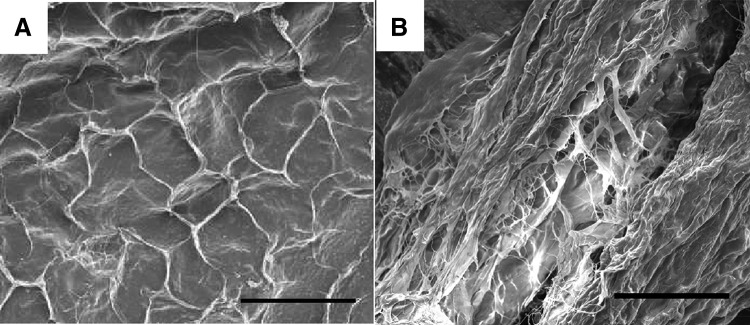
SEM images of the surface **(A)** and a cross-section **(B)** of DAFM/chitosan hybrid hydrogels. Scar bar: 100 μm. DAFM, decellularized AF matrix. SEM, scanning electron microscope.

**FIG. 2.**
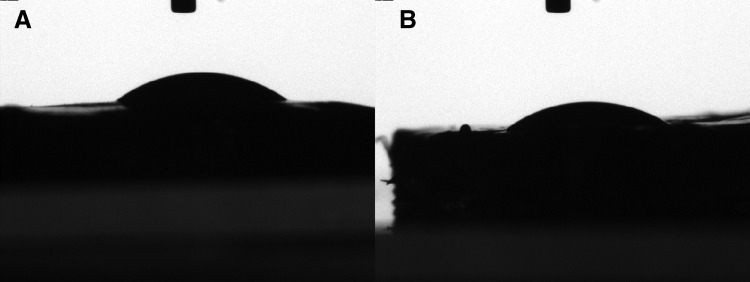
Contact angle diagram of DAFM/chitosan hybrid hydrogels **(A)** and DAFM/chitosan hybrid hydrogels with bFGF **(B)**. bFGF, basic fibroblast growth factor.

DAFM/chitosan hybrid hydrogels presented as porous three-dimensional networks through SEM image (Fig. 1). The contact angle of DAFM/chitosan hybrid hydrogels with and without bFGF 37.8° ± 1.4° and 36.1° ± 1.3°, respectively (*p* > 0.05; Fig. 2). The low contact angle for both hydrogels indicates high hydrophilia, which is propitious for AFSC adhesion.

### bFGF release kinetics

**FIG. 3.**
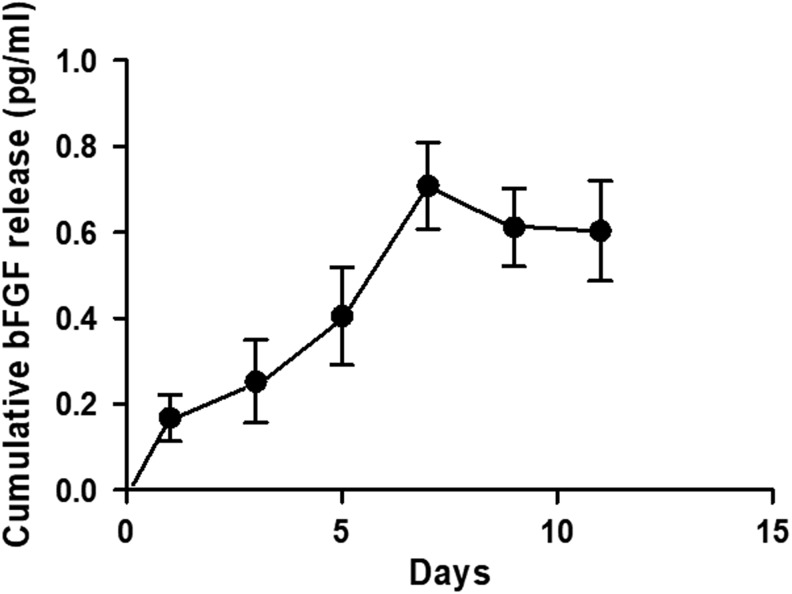
The bFGF release profile showed prolonged release of bFGF from DAFM/chitosan hybrid hydrogels over at least 1 week.

Cumulative release of bFGF from hydrogels reached a plateau after 7 days. This indicates that the DAFM/chitosan hybrid hydrogels could sustain continuous bFGF release for at least a week (Fig. 3).

### AFSC proliferation and morphology on hydrogels

**FIG. 4.**
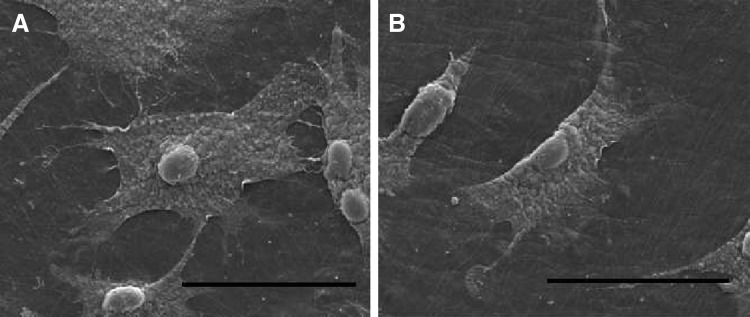
SEM images of AFSCs on DAFM/chitosan hybrid hydrogels **(A)** and DAFM/chitosan hybrid hydrogels with bFGF **(B)**. Scar bar: 50 μm. AFSCs, annulus fibrosus-derived stem cells.

**FIG. 5.**
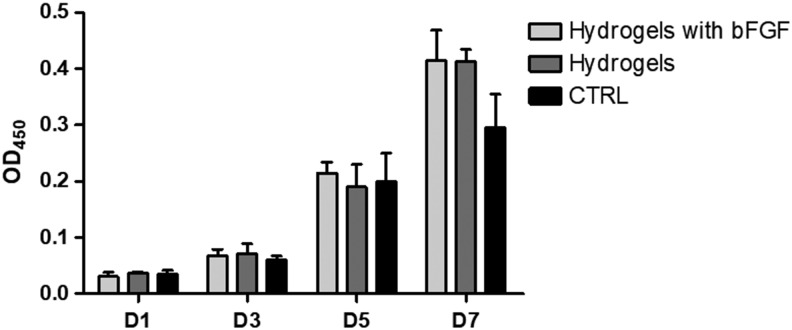
Cell proliferation of AFSCs on DAFM/chitosan hybrid hydrogels and DAFM/chitosan hybrid hydrogels with bFGF, cells growing on the TCPS as for control. TCPS, tissue culture polystyrene.

**FIG. 6.**
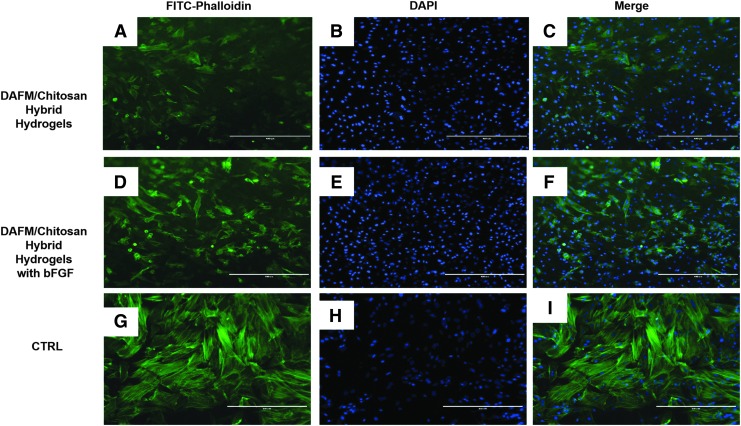
Cytoskeleton staining images of AFSCs for FITC-phalloidin (*green*) and DAPI (*blue*) on DAFM/chitosan hybrid hydrogels **(A–C)**, DAFM/chitosan hybrid hydrogels with bFGF **(D–E)** and on TCPS **(G–I)**. The scale bar represents 400 μm.

SEM imaging showed that AFSCs spread well on DAFM/chitosan hybrid hydrogels with and without bFGF (Fig. 4). CCK-8 assay results showed no significant differences between either type of hydrogel in terms of AFSC proliferation (Fig. 5). Cytoskeleton staining also indicated that AFSCs spread and proliferated well on both types of hydrogel (Fig. 6).

### AFSC gene expression on hydrogels

**FIG. 7.**
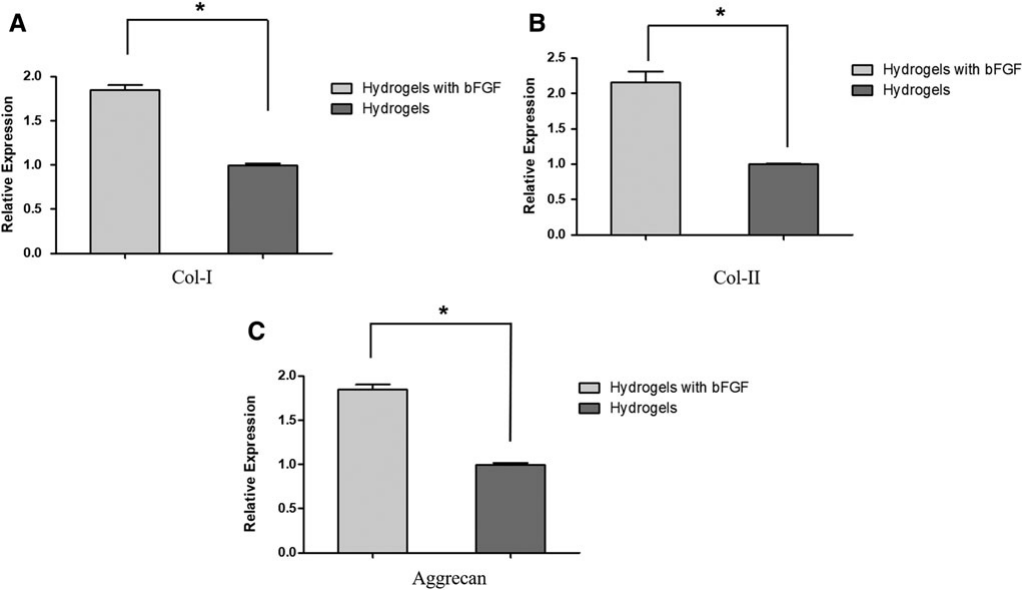
Gene expression of collagen-I **(A)**, collagen-II **(B)**, and aggrecan **(C)** of AFSCs cultured on DAFM/chitosan hybrid hydrogels and DAFM/chitosan hybrid hydrogels with bFGF for 2 weeks. *p < 0.05.

The level of expression of *collagen-I*, *collagen-II*, and *aggrecan* at 2 weeks of culture was twice as high in hybrid hydrogels with bFGF compared with hybrid hydrogels without bFGF (Fig. 7). The difference was found to be statistically significant (*p* < 0.05) for all three gene expression.

### ECM content

**FIG. 8.**
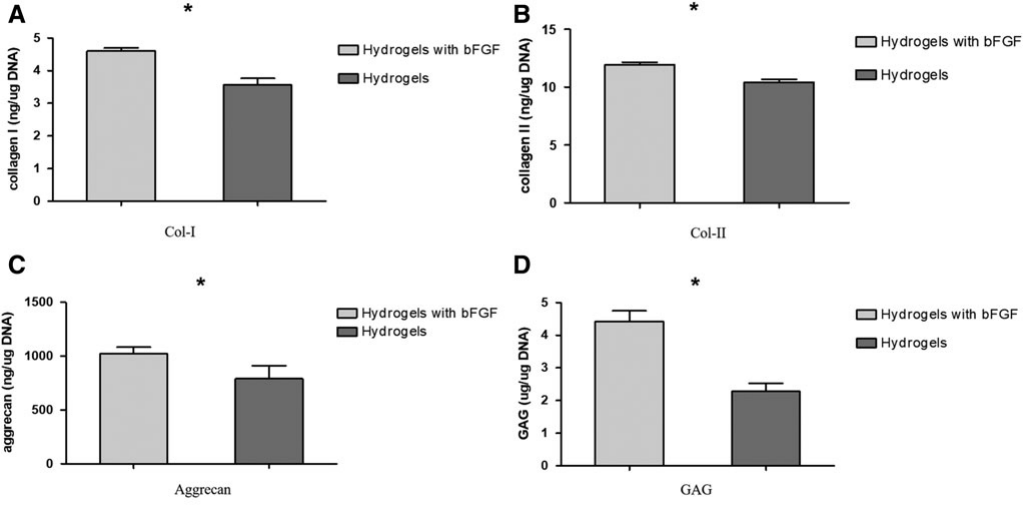
Collagen-I **(A)**, collagen-II **(B)**, aggrecan **(C)**, and GAG **(D)** production measured from DAFM/chitosan hybrid hydrogels and DAFM/chitosan hybrid hydrogels with bFGF normalized to DNA content, respectively, after culturing for 2 weeks.

Significantly more collagen-I, collagen-II, and aggrecan were released from AFSCs cultured for 2 weeks on DAFM/chitosan hybrid hydrogels with bFGF (4.68 ± 0.07 ng/μg DNA, 11.95 ± 0.19 ng/μg DNA, and 1022.23 ± 50.83 pg/μg DNA, respectively) than on those without bFGF (3.56 ± 0.16 ng/μg DNA, 10.42 ± 0.22 ng/μg DNA, and 790.95 ± 97.89 pg/μg DNA, respectively; all *p* < 0.05; Fig. 8A–C). The GAGs content released into the medium was also significantly greater for AFSCs cultured on hybrid hydrogels containing bFGF (4.43 ± 0.26 μg/μg DNA) than those without bFGF (2.28 ± 0.2 μg/μg DNA; *p* < 0.05; Fig. 8D).

## Discussion

In the present study, bFGF-releasing DAFM/chitosan hybrid hydrogels were successfully fabricated using genipin as a crosslinker. Moreover, these hydrogels were able to effectively support growth of healthy AFSCs and promote secretion of large quantities of ECM proteins, indicating their potential use as a scaffold for AF tissue engineering.

Seed cells play a central role in tissue engineering. However, application of AF cells^30^ and chondrocytes^31^ for AF tissue engineering is limited by their quickly changing phenotypes and decreased expression of collagen-II during monolayer expansion. On the contrary, adult MSCs have been reported to originate from tissues that preferentially differentiate into cell types residing in AF tissue, meaning that they tend to be tissue specific. Recently, we isolated and cultured rabbit AFSCs and found them to have a strong tendency to differentiate into AF-like cells relaying on the elasticity of scaffold.^32^ Therefore, we seeded rabbit AFSCs onto DAFM/chitosan hybrid hydrogel scaffolds in the present study to generate more AF-related ECM.

Decellularized matrices have been widely used in tissue engineering because they provide an *in vitro* microenvironment similar to that *in vivo.*^33,34^ Like many other decellularized matrices, the DAFM originates from native tissue, which reduces its immunogenicity after acellular treatment. This not only preserves the reticular structure closer to the actual AF tissue but also the secretion of growth factors and cytokines that regulate growth of seed cells. However, the mechanical properties of the DAFM are poor and subject to biological variations, such as host age, tissue source, and specific methods. Therefore, the present study examined whether incorporation of a complementary material could bolster DAFM suitability for AF tissue engineering. Not only does chitosan have good biocompatibility and strong mechanical properties but its molecular structure is also reported to be similar to an important proteoglycan component of AF tissue. For instance, Mirahmadi *et al.*^35^ found that glycerophosphate-chitosan hydrogels stimulated production of collagen-II and GAGs by chondrocytes *in vitro*.

Although hydrogels are promising candidates for engineering of soft tissues, such as cartilage, cardiac, and AF,^36,37^ and their hybridization enables different levels of mechanical strength and/or biochemical properties that are more specific to the target tissue. A study by Schek *et al.*^38^ using genipin-crosslinked fibrin gels demonstrated that their mechanical properties were similar to native AF tissue. In addition to being highly porous,^39^ the genipin-crosslinked DAFM/chitosan hybrid hydrogels fabricated in the present study also accommodated reliable AFSC adhesion and growth. Furthermore, the lack of significant difference between hybrid hydrogels with and without bFGF indicates that AFSC proliferation is largely dictated by the similarity to AF tissue structure and low contact angle of the hydrogel surface.

At present, AF tissue engineering failures have mostly been due to a lack of ECM protein production on scaffolds. Although the structure and components of scaffolds can affect ECM secretion by seeded cells, growth factors also play a very important role. Growth factors have been shown to regulate AF cells *in vitro*, and different growth factors have different anabolic effects on AF cells in both monolayer^40^ and three-dimensional cultures using agarose or alginate.^41^

In this study, bFGF was incorporated successfully into the scaffolds. As we expected, the gene expression of *collagen-I*, *collagen-II*, and *aggrecan* was absolutely upregulated with the help of bFGF. Furthermore, AFSCs from DAFM/chitosan hybrid hydrogels with bFGF could secrete more collagen-I, collagen-II, aggrecan protein, and GAGs than those on pure DAFM/chitosan hybrid hydrogels. Similar results were observed in the previous studies. Xu *et al.*^42^ concluded that bFGF could promote gene expression of rat MSCs from bone marrow, which were seeded on SF-CTGF/PLCL-PEO-FGF2 scaffolds. Shu *et al.*^43^ found that bFGF could significantly promote chondrocyte proliferation, and it increased GAGs and type II collagen synthesis.

Unfortunately, simply adding growth factors to the culture medium does not enable their regulation of seed cell metabolism long-term, especially considering delayed or modified release has been shown to be better for ECM synthesis than rapid, immediate release. For example, Takegami *et al.*^44^ proved that osteogenic protein-1 could increase proteoglycan production of NP and AF cells to regenerate degenerated discs. However, the use in treating disc degeneration is limited for its short biological half-life. In contrast, Li *et al.*^45^ investigated the addition of a binding structure by fusing a collagen-binding domain to IGF-1 and discovered that a significant increase in collagen-binding activity of the recombinant protein compared with IGF-1.

Methods of continuous, gradual growth factor release, however, remain a challenge. One approach to solving this problem involves incorporation of growth factors into the scaffold substrate. Several studies have investigated the potential of electrospun scaffolds for incorporation and release of growth factors. Sahoo *et al.*^46^ produced two types of poly(lactic-co-glycolic acid) nanofiber scaffolds incorporated with bFGF by blending electrospinning and coaxial electrospinning and showed that both released bFGF over 1 week. Similarly, Vadalà *et al.*^47^ synthesized a scaffold by electrospinning with direct incorporation of TGF-β1 into the poly(l-lactic acid) solution and found TGF-β1 could be measured after 4 days, with cumulative release reaching a plateau after 1 week.

In addition, hydrogels also have been used as drug carriers in AF tissue engineering. Previously, Gan *et al.*^48^ constructed a codelivery system involving a dextran/gelatin hydrogel with poly(lactic-co-glycolic acid) nanoparticles as carriers for controlled release of TGF-β3 for discogenic differentiation. Their release kinetics results indicated stable long-term release of TGF-β3 from day 1 to 28, which was attributed to their highly hydrophilic nature and good biocompatibility. In the present study, bFGF was successfully incorporated into DAFM/chitosan hybrid hydrogels and showed gradual release over 1 week, peaking in concentration after 7 days. The continuous release of bFGF by hybrid hydrogels was also found to significantly increase expression and secretion of collagen-I, collagen-II, aggrecan, and GAGs by AFSCs compared to hydrogels without bFGF incorporation.

There are limitations to the present study. AF tissue is a kind of complex fibrocartilage rather than homogeneous tissue; the inner and outer AF are characterized by different cellular phenotypes, biomechanical components, and mechanical properties. Our previous study separated the rabbit AF into three equally thick sections in the radial direction and found that the storage moduli from the outer to inner AF were different.^28^ However, the present study regarded the rabbit AF tissue as homogeneous and ignored the differences in mechanical properties, which could affect the performance of our DAFM/chitosan hybrid hydrogel *in vivo*. Therefore, future studies will include fabrication of DAFM/chitosan hybrid hydrogels with different moduli by adjusting the amount of genipin to better mimic native outer and inner AF tissue.

## Conclusions

The present study demonstrated that DAFM/chitosan hybrid hydrogels functionalized with bFGF were able to provide sustained and gradual release of bFGF as an anabolic stimulus for AFSC growth and secretion of important ECM factors. This indicates the potential of DAFM/chitosan hybrid hydrogels as a candidate scaffold for AF tissue engineering.
